# Using IVIM-MRI and R2⁎ Mapping to Differentiate Early Stage Liver Fibrosis in a Rat Model of Radiation-Induced Liver Fibrosis

**DOI:** 10.1155/2018/4673814

**Published:** 2018-12-03

**Authors:** Jianye Liang, Xiubao Song, Zeyu Xiao, Hanwei Chen, Changzheng Shi, Liangping Luo

**Affiliations:** ^1^Medical Imaging Center, The First Affiliated Hospital of Jinan University, Guangzhou, Guangdong, China; ^2^Department of Rehabilitation, The First Affiliated Hospital of Jinan University, Guangzhou, Guangdong, China; ^3^Department of Radiology, Panyu Central Hospital, Guangzhou, Guangdong, China

## Abstract

**Rationale and Objectives:**

To investigate the utility of intravoxel incoherent motion MRI (IVIM-MRI) and R2⁎ mapping in diagnosing early stage liver fibrosis in a radiation-induced rat model.

**Materials and Methods:**

Thirty rats were randomly divided into three groups with 10 rats in each group. Liver fibrosis was induced by exposure of right lobe of liver in each animal to 20 Gy of radiation. MRI examination was conducted at baseline, one month, two months, and three months after radiation using T1WI, T2WI, IVIM-DWI, and R2⁎ sequences. The pathological examination included hematoxylin eosin, masson trichrome, and prussian blue staining. D, D⁎, f, and R2⁎ values were measured in both left and right lobes for quantitative analysis.

**Results:**

Regarding the surviving 23 rats, eight rats were diagnosed with stage F0, ten with stage F1, and five with stage F2 liver fibrosis using METAVIR Scores. The D values of right lobes decreased (*P*<0.05), and R2⁎ values increased (*P*<0.01) significantly as fibrosis levels increased. But there was no statistical difference in D⁎ (P=0.970) and f values (P=0.079). R2⁎ value showed a strong positive correlation (r=0.819, P<0.001), while D value showed a negative correlation with fibrosis stages (r=-0.424, P<0.001). D⁎ (r=0.029, P=0.744) and f values (r=-0.055, P=0.536) were poorly correlated with fibrosis levels.

**Conclusion:**

IVIM-MRI and R2⁎ mapping are useful techniques for evaluating the severity of liver fibrosis in a radiation-induced rat model, and R2⁎ value is the most sensitive parameter in detecting early stage fibrosis.

## 1. Introduction

Liver cancer is one of the most fatal cancers in the world. The incidence rate and death rate due to liver cancer are increasing in recent years [1]. With widespread use of radiotherapy in the treatment of liver cancer, radiation induced liver injury (RILI) has become more common [2]. The pathological changes of RILI originate from an inflammatory response induced by radiation, then progress into liver fibrosis, leading to the development of cirrhosis. The progression of liver fibrosis can be suspended or even reversed if it is caught early and treated in time. As a result, an accurate and early diagnosis of liver fibrosis may help reduce the risk of radiotherapy-related complications and achieve a better prognosis [3]. Liver biopsy is regarded as the gold standard in the diagnosis of liver fibrosis. However, clinical adoption of liver biopsy has been limited due to poor repeatability, lack of localization, risks of bleeding and infection, and strong subjective bias in reading the results [4]. Therefore, clinicians try to find surrogate methods to diagnose liver fibrosis early and in a noninvasive manner. With recent advancement in magnetic resonance (MR) techniques, two novel sequences including intravoxel incoherent motion diffusion-weighted imaging (IVIM-DWI) and R2*∗* mapping, have been applied to detect the molecular and hemodynamic changes in various kinds of liver diseases.

IVIM-DWI is a sensitive method to visualize microscopic motion of water in tissue, including the molecular Brownian motion of water (D, pure diffusion) and the microcirculation perfusion (D*∗*, pseudo diffusion or perfusion). A feature of liver fibrosis is excessive synthesis of extracellular matrix, especially collagen fibers. Several previous studies have suggested that water molecular diffusion is restricted in fibrotic livers [5, 6]. On the other hand, since liver is an organ with large amounts of blood supply (approximately 25–30ml of blood per 100 g of liver), changes in liver perfusion may provide a good indicator of the fibrosis level [7].

R2*∗* mapping is another advanced technique, which measures the iron burden and deoxyhemoglobin levels in liver. Many recent studies suggest that iron is a paramagnetic substance that can shorten the relaxation time of T2 and T2*∗* [8–10]. Therefore, the transverse relaxation rate (R2*∗*=1/T2*∗*) is a noninvasive method for measuring the hepatic iron concentration, especially in liver fibrosis and cirrhosis [11].

So far, imaging and pathological studies have been used successfully to detect liver fibrosis in experimental animals, including biliary duct ligated and obstructive model, carbon tetrachloride (CCl_4_) induced model, and schistosomiasis induced model. However, studies of radiation-induced liver fibrosis are relatively uncommon. The progression, severity, and prognosis of above-mentioned models may be entirely different from radiation-induced liver fibrosis. Thus, it is important to investigate the pathophysiological mechanisms and imaging features of liver fibrosis in a radiation-induced rat model using techniques of IVIM-MRI and R2*∗* mapping.

## 2. Materials and Methods

### 2.1. Liver Fibrosis Model

Thirty female Sprague-Dawley rats weighing between 180 and 220 g were purchased from the experimental animal center of Guangzhou University of Chinese Medicine. The animal certificate number was SCXK (Guangdong) 2013-0034. The right lobe of each rat was irradiated with a fixed dose of 20 Gy at a source-skin distance of 100 cm using a VARIN 21-EX linear accelerator (Varian Medical Systems, America). The dose rate was 600 cGy/min and the radiation field was 2.5 × 2.5 cm. We used a lead plate of 5 cm to shield the area of left lobe when irradiated and regarded the left lobe as control group.

### 2.2. Experimental Procedure

All the rats (n=30) were taken for MR examinations before radiation. After irradiation, the irradiated rats were divided randomly into three groups of 10 each and examined by MRI at time points of one month, two months, and three months. In short, each rat received two MRI scans, one at baseline and one at monitoring. After scanning, the animals were sacrificed for pathological examination. The Institutional Animal Ethics Committee of Jinan University approved this protocol, and the Laboratory Animal Care and Usage Manual of the institute was strictly followed.

### 2.3. MRI Examination

Animals received intraperitoneal anesthesia with 2.0% pentobarbital sodium (0.2ml/100g) before imaging. Examination was performed on a 1.5-T Signa HDxt superconductor clinical MR system (GE Medical System, Milwaukee, WI). Each rat was placed in a high resolution wrist phased array coil and the abdomen was fixed to reduce respiratory motions.

MRI sequences were performed in an axial plane covering the liver. The parameters of T1-weighted imaging (T1WI) were set as follows: repetition time (TR) / echo time (TE) =420 / 10.6 ms, slice thickness / spacing = 3.0 / 0.5mm, matrix = 256 × 192, field of view (FOV) = 8 × 8cm, number of excitation (NEX) = 2. The parameters of T2-weighted imaging (T2WI) were set as follows: TR / TE = 2640 / 63.9ms, slice thickness / spacing = 3.0 / 0.5mm, matrix = 256 × 192, FOV = 8 × 8cm, NEX = 2. IVIM-DWI was acquired using single-shot spin-echo echo-planar-imaging with 11 b-values (0, 25, 50, 75, 100, 150, 200, 400, 600, 800, and 1000 s/mm^2^). The parameters were as follows: TR / TE = 4000 / 91.3ms, slice thickness / spacing = 3.0 / 0.5mm, matrix = 96 × 128, FOV = 10 × 5cm, NEX = 2. R2*∗* mapping was performed with three-dimensional spoiled gradient echo sequence and the following parameters: 16 TEs were used (TE=3.4, 9.3, 15.2, 21.2, 27.1, 33, 38.9, 44.8, 50.7, 56.6, 62.5, 68.5, 74.4, 80.3, 86.2, and 92.1ms), TR = 160 ms, FOV = 8.0 × 8cm, matrix = 160 × 160, and NEX = 2.

### 2.4. Image Analysis and Statistical Analysis

The post-processing of MRI data and measurement were carried out with MADC and R2Star software of Functool package on Advantage Workstation 4.5 version (GE Health Care, America). The biexponential model of IVIM-DWI was defined as

(1) $$\begin{array}{c} \frac{SI}{SI_{0}}=\left(\;1-f\;\right)\cdot \exp\left(\;-bD\;\right)+f\cdot \exp\left(\;-bD*\;\right) \end{array}$$

where *SI*_0_ is the mean signal intensity of the region of interest (ROI) at b = 0 s/mm^2^, and* SI* is the signal intensity at other b values. D represents the water molecular diffusion, which is also called true diffusion coefficient, D*∗* represents microcirculation perfusion, referring to pseudo-diffusion. F stands for perfusion fraction, which indicates the percentages of microcirculation perfusion in the overall diffusion effect [12, 13]. We fit the biexponential IVIM model using a segmented approach [14]. First, as b-value < 200 mm^2^/s is defined as low b-value, we assumed that the pseudo-diffusion is mainly reflected in this range and fit these data to the biexponential equation to obtain D*∗* and f values, whereafter all b-values >200 mm^2^/s data were fit to a mono-exponential equation to obtain the D value as the pseudo-diffusion is negligible in this range. We obtained the R2*∗* values using the Functool-R2Star software. The R2*∗* maps for each tumor were calculated by linearly fitting a single exponential model of the ln⁡(signal  intensity) to TE curve. The slope of ln⁡(signal  intensity) versus TE determines R2*∗* value. We registered the functional pseudo-color maps with T2WI for a better location. The ROI was defined to encompass a large homogeneous liver region without blood vessels and bile ducts. The same size (3mm^2^) and location of ROIs were then used for measuring the D, D*∗* f, and R2*∗* values. Relative T1 and T2 values were defined as the ratio of signal intensity of liver to muscular tissue in the same level on T1WI or T2WI.

Statistical tests were performed with software SPSS 13.0 (Chicago, IL). The quantitative results were expressed as mean ± standard deviation (SD). Kruskal-Wallis H and Mann-Whitney U tests were used to compare the parameters from different groups or pathological stages. Paired-samples* t* test was used to compare the difference between the irradiated right lobes and nonirradiated left lobes. The Spearman rank correlation coefficient (r) calculated the correlation strength between each parameter and fibrosis stages. A p-value < 0.05 was considered statistically significant. |r| ≥ 0.8 was considered a high correlation while 0.5 ≤ |r| < 0.8 a mild correlation.

### 2.5. Histological Analysis

After MR scanning, the rats were sacrificed by an intraperitoneal injection of 2.0% pentobarbital sodium. Livers were excised and fixed in 10% paraformaldehyde, then embedded in paraffin, and sectioned for histological examination. Hematoxylin eosin (HE), masson trichrome (MT), and Prussian blue (PB) staining were examined for structural changes, fibrogenesis, and iron deposition under a microscope. The fibrosis stages were scored by an experienced histopathologist (9 years of liver pathology experience) blinded to the MR results, using the METAVIR classification system [15]. Liver fibrosis was scored according to the following scale: F0 = no obvious fibrosis (dominated by inflammation), F1 = portal fibrosis without septa, F2 = portal fibrosis and a few septa, F3 = numerous septa without cirrhosis, and F4 = cirrhosis.

## 3. Results

### 3.1. General Information

Out of 30 rats, only 23 rats were studied for radiation induced liver fibrosis. During the study, 4 rats died within 48 h after irradiation due to intolerance to the irradiation, while 3 rats died during scanning because of anesthetic accidents. The numbers of rats in one-month, two-month, and three-month groups were eight, eight, and seven, respectively. The rats resumed normal activities and diets within two days after irradiation. No adverse effects were seen at the one-month time point. Decreased activity and logy response were observed two months later.

### 3.2. Histopathological Outcomes

**Figure 1:**
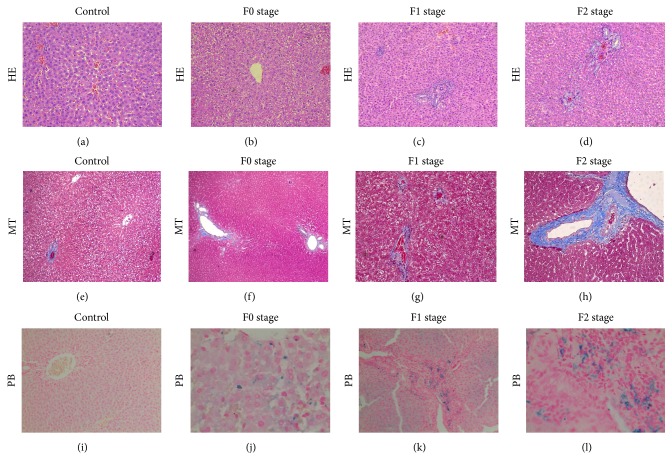
Pathological changes of left lobe (control) and right lobes at different fibrosis stages after radiation. Representative Hematoxylin eosin staining (HE, 20×), masson trichrome staining (MT, 20×), and prussian blue staining (PB, 20×) of liver sections. The lobular structure of left lobe is intact. The hepatic cord is orderly. The central vein is surrounded by the cords of hepatocytes which radiate out in all directions (a). Cytoplasm rarefaction and hepatocyte swelling can be seen in stage F0 (b). Infiltration of inflammatory cell and spotty necrosis could be seen in hepatic lobules at stage F1(c). Hyperplasia of small bile ducts and deposition of collagen fibers can be seen in portal areas at stage F2 (d). From stage F0 to F2, an increased amount of blue-stained fibrous tissues deposits in the portal areas and encompasses the central veins (f-h). No blue particles can be seen in the left lobe (i). An increased amount of blue-stained iron particles deposit is seen in the hepatocytes and macrophages from stage F0 to F2 (j-l).

Under HE staining, scattered spotty necrosis or focal necrosis within the hepatic lobules could be seen in the right lobes in the one-month group. Parts of portal areas were infiltrated by lymphocytes, but the lobular structures were intact. Hyperplasia of small bile ducts and deposition of fibrous connective tissues could be seen after two months. Three months later, in addition to previous manifestations, fibrous septa emerged and penetrated into the hepatic lobules, connecting adjacent portal areas and central veins. No abnormal histopathological changes could be seen in the control left hepatic lobes throughout the study (Figure 1).

Masson staining, which could stain the collagen fiber into blue-green color and had a greater contrast compared to HE staining, further verified the formation of fibrous tissues. Microscopically, proliferation of the blue-green collagen fiber could be found around the portal areas and central veins in the irradiated lobes. Fibrous septa could be seen in the three-month group (Figure 1).

Different amounts of deep-blue particles appeared in the hepatocytes and macrophages in all three groups using PB staining, which suggested the deposition of hemosiderin. In contrast, no blue particle was found in the left lobes (Figure 1).

Under the classification of METAVIR Scores, the distribution of fibrosis stage was as follows: F0 vs F1 vs F2 = 8 vs 10 vs 5. All the rats from the one-month group belonged to F0; the rats from the two-month group were scored as F1; five-sevenths from the three-month group belonged to F2, and two belonged to F1. Left lobes of all the rats were set as control groups.

### 3.3. Result of Conventional MRI 

**Figure 2:**
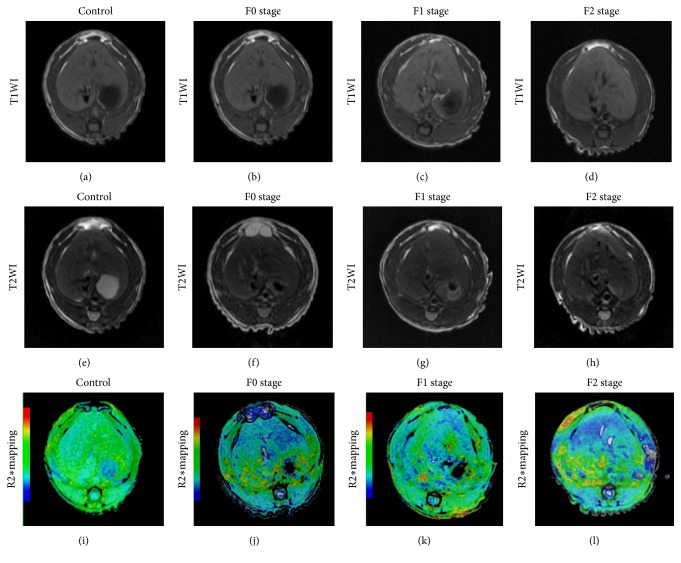
Axial T1WI, T2WI, and R2*∗* mapping at different fibrosis stages. a-d correspond to the rats without liver fibrosis, and the rats in stage F0, F1, and F2 on T1WI. e-h: on T2WI. The liver contour was smooth. The size, shape, and proportion of the liver are normal; no abnormal signal could be seen in both left and right lobes (a-h). (i-l) correspond to the rat without liver fibrosis, and the rats at stages F0, F1, and F2. The color ranging from blue to red represents the values ranging from low to high. No color difference between left and right lobe could be seen in the rats before radiation (i). R2*∗* values of the right lobes increase gradually from stage F0 to F2 (j-l).

T1WI and T2WI showed no obvious signal changes in the right lobes no matter before or after radiation. The signal intensity of right lobes was similar to that of left lobes at the same time points (namely, groups) (Table 1, Figure 2).

### 3.4. Evaluation of Liver Fibrosis Using IVIM-DWI and R2*∗* Mapping

Paired-samples* t* test showed that the D values in the right lobes were significantly lower than those in the left lobes one month (t=2.031, P=0.014), two months (t=3.218, P=0.005), and three months (t=4.356, P<0.001) after radiation. The R2*∗* values in the right lobes were significantly higher than those in the left lobes one month (t=2.436, P=0.021), two months (t=3.568, P=0.004), and three months (t=2.747, P=0.017) after radiation. Though D*∗* and f values of right lobes were slightly lower than those of left lobes two months and three months after radiation, the results were insignificant. The statistical results of D*∗* values one, two, and three months after radiation were t=0.873, P=0.405; t=1.790, P=0.087; and t=1.974, P=0.068. The statistical results of f values one, two, and three months after radiation were t=0.936, P=0.350; t=1.532, P=0.115; and t=1.879, P=0.085.

**Figure 3:**
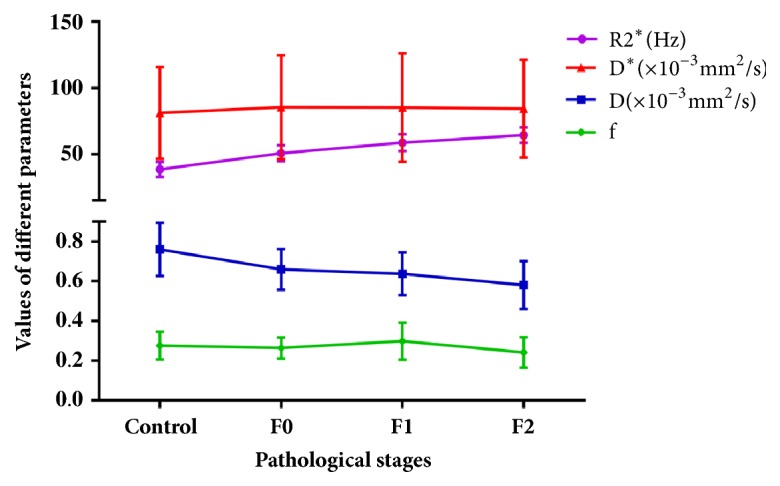
Trends of the four imaging parameters in the progression of liver fibrosis. D values decreased slowly as liver fibrosis progressed. R2*∗* values increased more dramatically after radiation. However, no significant changes could be seen in D*∗* and F values.

The comparisons of D*∗*, D, f, and R2*∗* values at different time points in right lobes were listed in Table 2. With regard to pathological stages, the D values of right lobes decreased significantly with the advance of liver fibrosis, but the changes in D*∗* and f values were not statistically significant (Table 3, Figure 3). Differences in R2*∗* values were significantly different between different pathological stages. The value increased as the pathological stages increased (Table 3, Figures 2 and 3). In addition, for each parameter the left lobe showed no statistically significant changes between different time points (Table 4).

### 3.5. Correlation Analysis between IVIM-DWI, R2*∗* and Pathological Stages.

The results of correlation test suggest that D values have a negative correlation (r=-0.424, P<0.001), whereas R2*∗* value has a positive correlation with the pathological stages (r=0.819,* P*<0.001), but there are no significant correlations for D*∗* and f values (r=0.029,* P*=0.744; and r=-0.055,* P*=0.536, respectively). The absolute value of correlation coefficient of R2*∗* value was the largest among the parameters mentioned above, which indicates a strong correlation with the pathological stages.

## 4. Discussion

Radiotherapy has become an integral part of adjuvant treatment of liver tumors. Liver fibrosis is relatively common in patients with hepatic radiotherapy. Although three-dimensional conformal and intensity-modulated radiotherapy [16] promise more accurate positioning and dose control, surrounding tissues may also be affected due to tumor heterogeneity and individual differences. For hepatobiliary malignancies, the radiation dose should be controlled in the range of 50 to 70 Gy [17]. The incidence rate of RILI is 5-10% when the total dose reaches 30-35 Gy [18]. The rates even increase greatly in patients who have a history of liver disease. There is no effective clinical treatment for RILI at present. If no timely intervention is taken, it will enter into the end-stage of liver cirrhosis. Early identification and timely adjustment of radiation doses are main approaches to suspend the progression of RILI. Compared with conventional MR sequences, the quantitative parameters such as D, D*∗*, f, and R2*∗* can reflect the biological information at a molecular level and have great advantages in the diagnosis of early stage of liver fibrosis.

In this study, no abnormal signal or statistical differences could be seen on T1WI and T2WI at different time points. This is different from the results seen in previous studies. The reason may be due to the modeling methods used in earlier studies. Previously, the liver fibrosis rat models were mainly induced by intraperitoneal injection of CCl_4_. Abnormal signal appeared on T2WI 48 hours after injection of CCl_4_. The entire liver was involved and a large area of necrosis was found in the hepatic lobules within two weeks [19]. Compared to liver fibrosis induced by CCl_4_, in the radiation-induced model the range was limited with relatively low severity and slow progression. HE staining confirmed the limited amount of pathologic changes in the right lobes. These differences may account for the incapability of conventional MR sequences in detecting early stage of fibrosis induced by radiation. Besides, the degree of liver fibrosis induced by regional radiotherapy was closely related to the level of radiation dose and the field used in the study. Geraci et al. [20] reported that approximately 50% of the rats exposed to 25 Gy or greater died, and most of these deaths occurred in one to two months after irradiation and showed evidence of damage to the stomach. In order to maintain a relatively high survival rate during research, we chose 20 Gy as the experimental radiation dose in this study. The 20 Gy dose of radiation was delivered in a single fraction at the beginning of study to induce an acute change of liver injury as many previous studies [21, 22] also established their radiation liver injury model by application of a single radiation dose ranging from 15 to 30 Gy. Clinically, multi-fractions are routinely used to treat a tumor in liver cancer radiotherapy practice as there are always a few of hypoxic tumor cells remaining in the tumor after a single radiation, which are insensitive to radiotherapy and become the source of recurrence. After a few days, the secondary irradiation will effectively kill the remaining tumor cells due to the reoxygenation of these hypoxic cells. Besides, the surrounding normal tissues will obtain enough time for reparation after radiation. However, if multi-fractions of 20 Gy were performed in our study, the incident rate of liver fibrosis in a rat model will be lower, the progression will be slower, and the severity of fibrosis will be milder due to the strong regeneration capacity of liver cells [23], which may increase the false negative probability of IVIM-DWI and R2*∗*mapping. As a result, a single full dose of 20 Gy is appropriate to induce a fibrosis model, as well as several animal studies manifested [22]. In the future, comparison with multi-fractions of radiation in a proper dose, which highly mimics clinical practice, may be a meaningful research direction.

In order to detect liver fibrosis induced by radiation early and accurately, more sensitive sequences are needed. IVIM-DWI and R2*∗* mapping show promise for early detection of liver fibrosis. In this study, D value decreased gradually with the progression of liver fibrosis. Besides, it could detect subtle changes as early as stage F0. In the early stages of liver fibrosis, a series of pathological changes could be found in hepatic lobules including swelling and degeneration of hepatic cells, infiltration by a large number of Kupffer cells and lymphocytes. The extracellular space was reduced and the diffusion of water molecule was restricted, resulting in a decrease in D values. After a few months, collagen fibers were increasingly generated by fibroblasts due to stimulation by growth factors and inflammatory factors. The fibers were deposited in the extracellular space and connected the portal areas and central veins, leading to further restriction of molecular diffusion. However, D value could not differentiate stage F0 from F1, and F1 from F2. The differences between these stages of liver fibrosis were insignificant.

D*∗* and f values are perfusion-related parameters which can reflect the hemodynamic changes of RILI quantitatively. The results revealed that the difference of D*∗* and f values between different pathologic stages was insignificant. Though HE staining demonstrated a certain amount of fibrosis deposited in the portal area and a few septa across the hepatic lobules, the central veins were not decreased significantly without obvious lobular deformation, even in stage F2. The blood flow from the interlobular artery and interlobular vein changed slightly or even cannot be detected by IVIM parameters. The study by Hu et al.[13] showed that D*∗* decreased and was negatively correlated with the severity of liver fibrosis. Decreased blood supply may occur due to structural changes of hepatic circulation at later stages of liver fibrosis (stages F2 and F3). Elevated resistance of hepatic sinus causes portal hypertension and a reduction in portal blood flow. The blood supply from the hepatic arteries tends to increase in order to offset the reduced blood flow from the portal vein. However, the increased blood flow fails to fully compensate for the reduction in portal blood flow, which causes a decrease in D*∗*. Besides, deposition and accumulation of collagen fiber in the hepatic parenchyma leads to an obstruction of the hepatic sinus, which is another reason for the decrease of D*∗* in a CCl_4_ induced rat model [24]. In a study of human liver fibrosis, Luciani et al. [6] found that values of D*∗* were significantly decreased in the cirrhotic liver group compared with the healthy liver group, but no statistical differences were seen in D and f values. The results were also inconsistent with the present study. This may be related to the subjects included in their study. Patients with chronic hepatic fibrosis showed obvious structural changes in the liver, which lead to a reduction in portal blood flow and D*∗* value.

In this study, R2*∗* value showed an upward trend from stage F0 to stage F2, and it correlated highly with liver fibrosis stages. This result was consistent with previously observed results [25]. R2*∗* value is closely related to iron deposition and deoxyhemoglobin concentration in the microenvironment [8]. In general, most of the iron is stored as ferritin or hemosiderin in the hepatocytes, followed by the Kupffer cells, cholangiocytes, and central venous endothelial cells [9, 26]. Hemosiderin has been found to be deposited in the Kupffer cells in an early stage of liver injury [27]. Blue-stained iron particles accumulate in the fibroblasts when the liver undergoes fibrosis. In the current study, PB staining confirmed the deposition of iron particles in the hepatocytes and Kupffer cells after radiation, and the blue particles increased gradually from stage F0 to stage F2. When ferritin and hemosiderin interact with surrounding hydrogen protons, the relaxation time of hydrogen protons is shortened, leading to a decrease in T2*∗* signal intensity and an increase in R2*∗* values [28, 29]. R2*∗* values would further increase as the RILI proceeds to fibrosis and iron deposition is aggravated. Notably, only a few iron particles are deposited in the liver at stage F0. However, R2*∗* values still increased significantly compared with control group. Insufficient hepatic blood flow causes microcirculatory disturbances and increase of deoxyhemoglobin at stage F0. Accumulation of deoxyhemoglobin, which is another paramagnetic material, may accelerate T2*∗* relaxation and cause an increase in R2*∗* values at the beginning of RILI [30]. Therefore, R2*∗* may be a valuable parameter in predicting the severity or stage of liver fibrosis* in vivo*.

We had not performed T1*ρ* sequence in this experiment as several studies had verified its value in liver fibrosis. T1*ρ* imaging technology is capable of assessing the activity of hydrogen proton exchange between free water and macromolecules in tissues and reflecting the metabolic information at a molecular level. The T1*ρ* value is mainly affected by the content of macromolecular substances, such as proteoglycan, sulphated-glycosaminoglycan, and collagen content. A different amount of proteoglycans, collagen, and other macromolecules were released and accumulated within the extracellular matrix when the liver proceeds into the fibrosis stage [19]. As a result, MR T1*ρ* relaxation may have a superiority in detecting these molecular changes in liver fibrosis. However, it cannot denote the exact macromolecules changed during the disease progression. Besides, it cannot provide further information regarding hemodynamics, cell viability, oxygen metabolism, and iron content, which are also features of liver fibrosis. In the future, we will compare the diagnostic performance of T1*ρ* sequence with IVIM-DWI and R2*∗* mapping in diagnosis of liver fibrosis.

There were some limitations in this study. First, the exact radiation dose and exposure field used are hard to ensure in a rat model. The progress and severity of liver fibrosis induced by radiation in a rat model may not be reflective of human liver fibrosis. Second, deposition of iron particles was only confirmed using PB staining. Other paramagnetic materials such as deoxyhemoglobin were not confirmed by hematological examination. Third, there were no stages F3 and F4 RILI found in this study and the differences between early stage and advanced stage liver fibrosis could not be seen. Additional study with a larger radiation dose and a longer observation time may be able to address this issue.

## 5. Conclusions

Our study showed that IVIM-MRI and R2*∗* mapping are two valuable and noninvasive techniques in evaluating the stage and severity of liver fibrosis* in vivo*. D reflects the molecular changes in the microenvironment while R2*∗* reflects iron deposition and blood-oxygen level in early stages of liver fibrosis. Besides, R2*∗* value is more sensitive and relevant than D value and is expected to be a useful surrogate indicator in detecting early stage of liver fibrosis.

## Figures and Tables

**Table 1:** Relative T1 and T2 values of left and right lobes at different time points.

	base	1m	2m	3m	*χ* ^2^ (*P)*
T1 of right lobes	1.38 ± 0.07	1.33 ± 0.07	1.27 ± 0.12	1.43 ± 0.13	5.72 (0.13)
T1 of left lobes	1.38 ± 0.07	1.24 ± 0.12	1.29 ± 0.12	1.40 ± 0.16	6.94 (0.07)
*Z value (P)*	0.97 (0.33)	1.60 (0.11)	1.07 (0.29)	1.10 (0.27)	—
T2 of right lobes	1.04 ± 0.20	1.10 ± 0.23	0.96 ± 0.03	1.22 ± 0.08	4.27 (0.23)
T2 of left lobes	1.04 ± 0.17	1.12 ± 0.17	1.01 ± 0.07	1.23 ± 0.13	4.30 (0.27)
*Z value (P)*	0.36 (0.72)	0.54 (0.59)	1.07 (0.29)	0.73 (0.47)	—

note: base, 1m, 2m, and 3m denote the groups before and one, two, and three months after radiation.

**Table 2:** Comparison of D*∗*, D, f, and R2*∗* values at different time points in right lobes.

	D*∗* (×10^−3^mm^2^/s)	D (×10^−3^mm^2^/s)	f	R2*∗* (Hz)
Base (n=30)	80.3 ± 75.9	0.684 ± 0.174	0.275 ± 0.079	36.43 ± 7.64
1m (n=8)	81.7 ± 74.2	0.652 ± 0.141	0.273 ± 0.064	45.64 ± 6.48
2m (n=8)	77.4 ± 56.2	0.621 ± 0.231	0.268 ± 0.058	50.46 ± 5.45
3m (n=7)	83.1 ± 45.8	0.579 ± 0.166	0.276 ± 0.039	59.73 ± 8.44

*P*	0.503	**0.027**	0.096	**0.019**

note: units of ADC, D*∗*, D were mm^2^/s, f was percentage. base, 1m, 2m, 3m were stood for groups of pre-irradiation and post-irradiation for one, two, three months.

**Table 3:** Comparison of D*∗*, D, f, and R2*∗* values at different pathological stages in the right lobe.

	D*∗* (×10^−3^mm^2^/s)	D (×10^−3^mm^2^/s)	f	R2*∗* (Hz)
Control group	81.2 ± 34.8	0.760 ± 0.133	0.276 ± 0.070	38.42 ± 5.69
Stage F0 (n=8)	85.4 ± 39.2	0.660 ± 0.103	0.264 ± 0.053	50.75 ± 6.12
Stage F1 (n=10)	85.2 ± 41.0	0.637 ± 0.108	0.298 ± 0.094	58.73 ± 6.40
Stage F2 (n=5)	84.4 ± 37.1	0.581 ± 0.121	0.241 ± 0.077	64.34 ± 5.87

*P*	0.970	**0.001**	0.079	**0.001**

**Table 4:** Comparison of D*∗*, D, f, and R2*∗* values at different time points in left lobes.

	D*∗* (×10^−3^mm^2^/s)	D (×10^−3^mm^2^/s)	f	R2*∗* (Hz)
Base (n=30)	77.8 ± 27.4	0.719 ± 0.127	0.269 ± 0.058	37.77 ± 4.90
1m (n=8)	78.2 ± 33.4	0.704 ± 0.153	0.279 ± 0.081	37.10 ± 5.25
2m (n=8)	79.0 ± 36.9	0.737 ± 0.125	0.270 ± 0.088	37.64 ± 6.11
3m (n=7)	84.6 ± 36.4	0.749 ± 0.182	0.283 ± 0.079	38.24 ± 4.92

*P*	0.689	0.363	0.745	0.958

note: base, 1m, 2m, and 3m denote the groups before and one, two, and three months after radiation.

## Data Availability

The data used to support the findings of this study have not been made available because this study was involved in another experiment which was inappropriate to present the data currently.
